# Autism and the Sensory Disruption of Social Experience

**DOI:** 10.3389/fpsyg.2022.874268

**Published:** 2022-07-25

**Authors:** Sofie Boldsen

**Affiliations:** Department of People and Technology, Roskilde University, Roskilde, Denmark

**Keywords:** autism, qualitative research, sensory differences, social interaction, embodiment, phenomenology

## Abstract

Autism research has recently witnessed an embodied turn. In response to the cognitivist approaches dominating the field, phenomenological scholars have suggested a reconceptualization of autism as a disorder of embodied intersubjectivity. Part of this interest in autistic embodiment concerns the role of sensory differences, which have recently been added to the diagnostic criteria of autism. While research suggests that sensory differences are implicated in a wide array of autistic social difficulties, it has not yet been explored how sensory and social experience in autism relate on a phenomenological level. Given the importance of the sensory dimension of social encounters in phenomenological analyses of autism, this question must be considered crucial. This article investigates the role played by sensory differences in autistic social experience. Through a phenomenological analysis informed primarily by the philosophy of Maurice Merleau-Ponty with particular emphasis on the relation between intersubjectivity and perception, I argue that sensory differences affect the way other people appear in autistic experience on a pre-reflective level. By drawing on autistic young adults’ experiential descriptions of social encounters, this article identifies three aspects of how sensory differences affect social experiences in autism. First, social encounters manifested as sensorially disturbing, chaotic, and unpredictable events. Second, the embodied expressions of others appeared unfamiliar, threatening, and promoted a sense of detachment from the social world. Third, deliberate practices were employed to actively seek perceptual and social meaning in these disorienting social encounters. This analysis stresses the importance of understanding embodied intersubjectivity through its sensory dimensions. In addition, it indicates an important avenue for future research in exploring the potential role of practice in maintaining an intuitive grip on social meaning. By approaching social encounters as sensory and perceptual events, I emphasize how social difficulties in autism are inherently world-involving phenomena rather than a cognitive deficit reducible to the autistic person.

## Introduction

Autism is a developmental disorder characterized by pervasive difficulties with social communication and interaction and restricted, repetitive interests and behaviors (Kanner, 1943; Asperger, 1991; American Psychiatric Association, 2013; World Health Organization, 2018). Since the 1980s, the paradigm of theory of mind has retained a dominant position in autism research and described social difficulties in autism as results of a failed maturation of the cognitive system arguably responsible for our ability to infer the mental states of others (Baron-Cohen et al., 1985). Apart from the theory of mind hypothesis, major theories of autism include the executive dysfunction theory, according to which autistic persons have a weakened ability to manage their cognitive processes (Ozonoff et al., 1991), and the weak central coherence theory, which focuses on a detail-oriented processing style as compromising the ability of autistic persons to process global and contextual information (Happe and Frith, 2006). Despite their differences, these major theories represent an approach to autism focused on cognitive functioning. One prominent idea in the theory of mind literature is that autistic persons lack access not only to others mental states’ but also to their own (Frith and Happe, 1999). Following such a framework, it makes little sense to explore subjective experience in autism, and as has been argued, autism research has historically not paid much attention to and even discredited autistic accounts of their own experiences (McGeer, 2005; Milton, 2014b; Botha, 2021). The starting point of this article is the assumption that the most important source of knowledge about autism is the experiences and perspectives of autistic persons.^1^ For years, autistic persons have been pushing for an acknowledgment and understanding of sensory and bodily aspects of autism, which have been neglected in a paradigm where the cognitive machinery is assumed to constitute the essence of autism [see Milton (2014a); Leong (2016) and Walker (2019)].^2^

Based on a recognition of subjectivity and embodiment in autism, emerging phenomenological approaches have also objected to the predominantly cognitive paradigm in autism research and suggested a reconceptualization of autism as a disorder of embodied intersubjectivity rather than higher-order cognitive processes (Gallagher, 2004; Zahavi, 2005; Fuchs, 2015; Krueger, 2021). As argued by Thomas Fuchs, “what autistic children primarily lack is not a theoretical concept of other minds but a primary sensus communis or a sense of bodily being-with-others” (Fuchs, 2015, p: 198). Indeed, autism research has witnessed an embodied turn, partially precipitated by a growing body of empirical research demonstrating the role of pervasive sensorimotor differences in the development and manifestation of autism (Hobson, 2002; Fournier et al., 2010; Donnellan et al., 2013; Eigsti, 2013; Robertson and Baron-Cohen, 2017).^3^

Part of this increased orientation toward bodily processes in autism is a renewed interest in sensory differences, which were recognized already in the early years of autism research (Kanner, 1943; Hutt et al., 1964; Hermelin and O’Connor, 1970; Ornitz, 1974). Despite having been largely forgotten as the history of autism research progressed, recent research suggests that sensory differences occur in over 90% of autistic individuals and impact fundamental areas of functioning, such as perception, action, engagement in everyday activities, and social interaction (Leekam et al., 2007; Baranek et al., 2008; Robertson and Baron-Cohen, 2017). In addition, the autism literature suggests that sensory differences are implicated in a wide array of social difficulties in autism (Woynaroski et al., 2013; Dakopolos and Jahromi, 2019; Kuno-Fujita et al., 2020; Lin, 2020). Autistic self-reports have highlighted the importance of subtle and pervasive sensory differences for understanding social difficulties (Cesaroni and Garber, 1991; Williams, 1992; Hale and Hale, 1999; Biklen, 2005; Grandin, 2006). For example, a 13-year-old boy, “Albert,” describes being overstimulated by touch, reporting that “it hurts” and “it’s too much,” causing him to stiffen up in situations involving physical contact (Cesaroni and Garber, 1991, p: 306–307). Autistic writer and artist Donna Williams explained how sensory differences affected her ability to process information through multiple sensory modalities, causing her to “turn off her ears” if she needed to maintain eye contact (Williams, 1992). While such issues undoubtedly affect social engagement in autism, the question remains of how to characterize this process further and how to understand it on an experiential and phenomenological level.

This article aims to clarify the relationship between sensory and social experience in autism by exploring how the embodied expressions of other people in social contexts appear in autistic sensory experience. Given the importance granted to the sensory and perceptual dimension of social encounters by phenomenological accounts of autism, this question must be considered crucial. To shed light on this question, I draw on qualitative data from an ongoing research project on autistic social experience and practice gathered through extensive fieldwork in social groups for adolescents and young adults with autism and in-depth qualitative interviews with the group participants.

Through a phenomenological analysis informed primarily by the philosophy of Maurice Merleau-Ponty, I argue that sensory differences affect the way other people appear in autistic experience on a pre-reflective level. By exploring the diverse ways in which sensory differences can destabilize the experience of social encounters, this article points to the importance of understanding the sensory dimensions of embodied intersubjectivity and the potential role of practice and activity in the experience of social meaning. By approaching social encounters as sensory and perceptual events, I emphasize how social difficulties in autism are closely related to the experience of the surrounding world and thus inherently world-involving phenomena rather than cognitive deficits reducible to the autistic person.

## Materials and Methods

### Theoretical Background: Intersubjectivity and Perception in Merleau-Ponty

A central aspect of phenomenological accounts of autism is the attention to the embodied and perceptual nature of intersubjective encounters. Below, I will introduce a phenomenological understanding of the relation between intersubjectivity and perception, which will form the theoretical basis of the following analysis of sensory and social experience in autism. The phenomenological approach to social experience has much in common with more recently developed second-person approaches to social cognition and interaction, according to which social understanding proceeds directly in concrete interactional contexts without the need for inference or mental representation. Indeed, the Merleau-Pontian and Husserlian notion of the body has contributed significantly to developing enactive accounts of cognition (Gallagher, 2018; Bar, 2020). Despite this common ground, the following account will remain largely within the parameters of Merleau-Ponty’s phenomenology to pursue and focus on the experiential and perceptual features of intersubjectivity rather than on the interactional dynamics themselves.

On a phenomenological account, social understanding and interaction are not processes mediated by reflective activity, as the experiential life of another person is present immediately in the form of bodily expressivity. According to Merleau-Ponty, observable behavior does not hide the other’s emotions, intentions, thoughts, and desires as inaccessible internal states (Merleau-Ponty, 1964a, p: 52–53). Instead, they are embodied as meaningful expressions directly available in perceptual experience.^4^ Social encounters are thus perceptual events, which Merleau-Ponty emphasizes through his description of the “esthesiological phase” of empathy:

The whole riddle of Einfühlung lies in its initial, “esthesiological” phase; and it is solved there because it is a perception. He who “posits” the other man is a perceiving subject, the other person’s body is a perceived thing, and the other person himself is “posited” as “perceiving.” It is never a matter of anything but co-perception (Merleau-Ponty, 1964b, p: 170).

Drawing on Edmund Husserl’s analysis of double sensation (Husserl, 1989), Merleau-Ponty argues that the experience of others is a perception in which the other’s body appears not only as a perceived thing but a perceiving, sensing, living being. One perceives the other as perceiving, and consequently, the other appears in the form of a different sensibility and mode of perception.

Recently, Køster (2021) has drawn attention to this sensory dimension of intersubjectivity in his analysis of the felt sense of the other. For example, hearing the unique sound, melody, and tonality of the voice of a loved one or noticing their distinct scent or patterns of movement represents how the experience of others is sensory and affective rather than cognitive or epistemic. Drawing primarily on Merleau-Ponty, Køster describes the felt sense of the other as a sensorium, understood as an experiential structure constituted reciprocally by the other’s expressive style and the perceiver’s style of perceiving (Køster, 2021, p: 64).

The sensorium of the other thus denotes a complex phenomenological structure through which the other person appears “as a unified whole through a range of sensory modalities” (Køster, 2021, p: 58). Køster outlines the sensorium of the other as an inter-modal phenomenon through the ability of sensory modalities to “coalesce and manifest in highly intermodal and synesthetic ways” (Køster, 2021, p: 67). Køster’s analysis aligns with Merleau-Ponty’s understanding of social experience as revealing the other as a meaningful whole rather than a series of impressions. According to Merleau-Ponty, perception always transcends what is directly given in perception as the object as a totality always gives more than its intuitively given profiles. In the following, Merleau-Ponty describes how perception is thus co-constituted by the pre-reflective co-intention of a horizon of non-perceptions.

The perceived is composed of lacunae that are not merely “non-perceptions.” I can know that a crystal that I see or touch has a “uniform” shape without having, even tacitly, counted its sides. I can become familiar with a person’s face without ever having perceived, for itself, the color of the eyes (Merleau-Ponty, 2012, p: 11).

Although the world is always given perspectivally rather than in its totality, objects appear in experience as meaningful wholes. According to Merleau-Ponty, “we hardly perceive any objects at all, just as we do not see the eyes of a familiar face, but rather its gaze and its expression” (Merleau-Ponty, 2012, p: 294). In this way, the experience of another person relies on only tacitly (rather than thematically) experiencing aspects such as facial features. Thus, perception of objects and people both rely on this oscillation between object and horizon that Merleau-Ponty characterizes as the “two-sided act” of perception.

To see the object is to plunge into it and because objects form a system in which one object cannot appear without concealing others. More precisely, the inner horizon of an object cannot become an object without the surrounding objects becoming an horizon, and so vision is a two-sided act (Merleau-Ponty, 2012, p: 70).

Merleau-Ponty’s idea of vision as a “plunging into” objects furthermore emphasizes how perception is active communication with, rather than something “exerted on,” the world (Merleau-Ponty, 2012, p: 53). In their phenomenological analysis of the notion of sensus communis, Samuel Thoma and Thomas Fuchs emphasize how perception is a way of “sensing and touching the world, whereby the subject gets in close contact with what is sensed, moves towards it, and is moved by it” (Thoma and Fuchs, 2017, p: 139). This reciprocity of perception is perhaps most clearly expressed in the perception of another person, where we experience the encounter with another perspective on the world in which “a different comportment and a different gaze take possessions of my things” (Merleau-Ponty, 1964b, p: 170).

This understanding of the perceptual dimension of social encounters provides a sound theoretical framework for understanding how sensory differences affect social experience in autism through facilitating attention to the complex perceptual and bodily structures of our experience of other people. In the following analysis, I will focus primarily on Merleau-Ponty’s notion of horizon and Køster’s notion of the felt sense of the other as a conceptual basis for exploring the variety of ways autistic persons sense, feel, and handle social encounters.

### Methodology

The following analysis draws on empirical data from an ongoing qualitative and phenomenological study exploring the pre-reflective and sensory aspects of social experience in autism and its connection to embodied and material practices. This phenomenological orientation warrants an approach to qualitative research adapted to how the phenomenon in focus expresses itself in experience (Boldsen, 2021). This study draws from resources from both descriptive phenomenological psychology (Giorgi, 2009; Englander, 2020; Englander and Morley, 2021), micro-phenomenology (Petitmengin, 2006), and interdisciplinary approaches to phenomenology and qualitative science (Høffding and Martiny, 2016; Ravn, 2021). The empirical context in which this study explored social experience and practice are social groups for young people with autism. These groups represent an increasingly popular approach to addressing social difficulties in autism by facilitating friendship between peers and providing social competence training.

This study employed an exploratory research design that combined ethnographic fieldwork and qualitative interviews within a phenomenological framework. Fieldwork spanned one and a half years, from October 2017 to June 2019, and included participant observation in two social groups for autistic adolescents and young adults hosted by a Danish autism center: one for autistic women aged 18–27 and one mixed-gender group for autistic young people aged 15–21. Observations at the biweekly group meetings were occasionally supplemented with day trips or sleepovers during weekends and after-meeting debriefings with the autism center staff. In addition, qualitative interviews were conducted from February 2019 to May 2019 with eleven group participants, seven from the youth group and four from the women’s group, one interview per participant. The length of the interviews ranged from fifty to ninety minutes.

In two ways, fieldwork in the autism groups played a crucial role in supporting the qualitative interviews that formed the second part of this study’s data collection. First, by providing access: As group participants tended to distrust psychologists and the possibility of evaluation, and because face-to-face interactions can feel unsafe for autistic individuals, prolonged fieldwork eased the process of building the interpersonal trust necessary to conduct qualitative interviews. Second, by refining the focus of interviews: Emerging familiarity with the group participants’ experiences and social practices pointed to relevant topics to address in conversation and helped the interviewer’s participation in the process of recalling and describing various experiences (Høffding and Martiny, 2016; Ravn, 2021).

A phenomenological approach to qualitative interviews was adopted to pursue experiential, pre-reflective, and sensory features of social engagement in autism. This approach drew inspiration from the interview techniques advocated by phenomenological psychology (Englander, 2020) and the micro-phenomenological interview (Petitmengin, 2006). Using qualitative interviews within a phenomenological framework requires the facilitation of experiential descriptions rather than explanations or opinions (Høffding and Martiny, 2016; Englander, 2020). This requirement was cashed out by focusing interview questions on concrete instances of social experience and working through its pre-reflective and sensory features (Petitmengin, 2006). A semi-structured interview guide was developed that focused on concrete social situations and the sensory, bodily, and material features constituting negative and positive social experiences. Many aspects of interviewing may be difficult for autistic persons. Social interaction with the interviewer may be experienced as overwhelming, verbalization of experiences and emotions may be difficult, and the interview situation itself may be stressful and anxiety-provoking. In addition, autistic persons express themselves in atypical ways, motivating some scholars to recommend visual aids during the interview (Shepherd, 2015). To accommodate such challenges, all interviews were conducted at the autism center during group nights to meet participants’ need for stability and sense of security and minimize intervention into everyday life routines. Great care was taken to produce an autism-friendly interview situation. Interviewees were invited to engage in whatever activity they preferred, such as drawing, fidgeting, and listening to music during the interview to increase a sense of comfort in a potentially stressful situation. In addition, an employee from the autism center with whom interviewees felt safe was made available for debriefing after the interviewee if needed. After each interview and in dialogue with developing understandings of autistic social practices achieved during fieldwork, the interview guide was refined and specified as new aspects of social experience in autism emerged.

The analysis in this study aimed to explicate and describe the phenomenological structures underlying and supporting the autistic social practices and experiences documented in the data. This aim was reached through a method of analysis that employed both exploratory and phenomenological strategies divided into two stages. The first analytic stage sought to understand the lived experiences and practices expressed in the data on their own premises. This process was initiated by exploratorily reading and re-reading the data material, following Amedeo Giorgi’s strategy (Giorgi, 2009). Through this initial immersion, codes were developed to organize data according to central aspects of participants’ lived experiences of social interactions, such as “being overwhelmed by voices” or “disconnection from others.” The second analytic stage described the phenomenological structures supporting the participants’ lived experiences and practices. Two processes defined this stage. The practice of explicating latent or tacit meanings in the data (Englander and Morley, 2021) and exploring structural aspects of these meanings with inspiration from phenomenological analyses and concepts (Ravn, 2021). At this stage, the coding of the data aimed to connect participants’ experiences with phenomenological themes reflecting the analytic interest of the researcher, such as “reciprocity” and “foreground and background in sensory experience.” In the following presentation, data excerpts were selected according to their ability to exemplify core features of both analytic stages. Moreover, the presentation will reflect the process of moving from how participants describe and understand their own experiences to a discussion of the phenomenological structures underlying and supporting these experiences.

The Danish Committee System on Health Research Ethics reviewed the ethical dimensions of his study. Following the guidelines on research ethics in the social sciences and humanities provided by the European Commission, the following steps were taken to ensure the ethical integrity of this study (European Commission, 2018). First, informed consent was sought during fieldwork and at the time of interviewing, where gradually increased familiarity between researcher and research participants formed the basis of trust. All research participants and their parents, if underage, signed a written consent form. Second, data were treated following the EU General Data Protection Regulation [Regulation (EU) 2016/679 of the European Parliament and of the Council, 2016], pseudonyms for the research participants were used, and place names and other details enabling the identification of research participants were changed.

## Results

### The Sound of Voices and the Texture of Skin

To describe how other people appear in autistic sensory experience, I will present a series of descriptions of social experiences provided by adolescents and young adults with autism during qualitative interviews. As described above, interviews addressed how research participants experience social interaction, why it can be challenging, and how they handle these challenges. As examples of socially challenging situations, participants described a wide range of social situations, such as family dinners, small talk with friends, encountering strangers, parties, or group exercises at school. A general feeling of being overwhelmed by and separated from others in social situations runs through these descriptions. One prominent feature of these experiences is their sensory manifestation. Participants usually described being overwhelmed as the uncomfortable sensory and affective experience of sound, touch, or visual aspects of the social environment appearing oppressive, chaotic, and threatening. In addition, social situations were described as intrusive and claustrophobic and as provoking a form of withdrawal or retreat from interactions with others and a sense of being separated from one’s surroundings and other people.

Johanne, a 26-year-old woman with Asperger’s Disorder, describes this feeling of being overwhelmed in a social setting through her experience of the buzzing soundscape of voices and conversations at a family gathering:

It is like a constant blanket of sound that just keeps coming at you until you are totally disoriented. […] You cannot really get away, and it’s like a sea, that just… It’s just everywhere, and you cannot get away. […] I do not know if the sounds, in a way, are more penetrating… As if they are reaching a deeper layer of the psyche or that they do not just pass by.

Johanne describes the surrounding voices as a sea or a blanket of sound rushing over her in an invasive or penetrating manner. The social gathering she is part of appears sensorially dense and saturated to a point where the conversations around her do not consist of meaningful voices but dissolve into undecipherable noise. Nina, a 17-year-old woman with Asperger’s Disorder, describes a similar experience of sensory saturation in a situation in which she is trying to have a conversation at a party:

There were many people whirling around me, so there were a lot of impressions, also because I wanted to look at and hear and follow everything. […] It’s like if you are trying to focus on something, and then there is someone in the periphery of your eyesight flicking their fingers [showing a flicking/fanning movement with the fingers beside her head], and it just will not stop. When I was sitting at a table talking to someone, then that was what those dancers that whirled around felt like. […] It’s a bit like if someone is coming up behind you […] Almost as if you can feel someone’s touch without them actually touching you.

Like Johanne, Nina describes how the sensory background of a social situation, here the movements of dancing people in her visual periphery, refuses to remain a tacit background and demands attention with acuteness and emergence. In Johanne’s case, the auditory horizon of casual conversation around the table refused to remain a tacit background. Instead, it intensified and experientially ‘thickened’ until it was no longer background but an overwhelming ‘sea’ of noise. Simultaneously, everything and nothing are in focus, thus representing an imbalance between the thematized and the tacitly co-intended in perceptual experience. Nina’s description of the peripheral whirling dancers elaborates on this imbalance. In this case, it seemed that the dancers’ movements did not remain visually implicit but demanded attention as explicit objects and disturbed her experience of proximity and presence to the conversation in which she was engaged.

There is a sense in autistic social experience that various co-perceived aspects, such as gestures and movements in the background, or the acoustic qualities of a voice, lose their tacit and implicit character and surface in perception as thematized objects of attention. This implies a double movement. First, the usual background noise in the auditory and visual environments loses its character as an unnoticed background and instead moves to the foreground of experience. Second, the sense of presence in the concrete interaction dissolves into a sea of noise and movement. Thus, the perceptual saturation implied in the above descriptions indicates an experiential disconnection from the perceptual situation and its meaning.

What is described by Merleau-Ponty as the two-sided act of perception seems to manifest radically differently in these descriptions. Rather than the smooth oscillation between object and horizon that Merleau-Ponty describes, autistic social experience is described by Nina and Johanne as a perpetual foregrounding or coming-to-attention of the world. Rather than remaining a background upon which the embodied expressions of the other person can stand out as meaningful, the sensory surroundings are experienced as invasive and violently closing in. The idea of perception as a simultaneous sense of moving and being moved by the world is replaced by a sense of being pushed away by a sensorially chaotic, unpredictable, and unfamiliar world.

In another context, Nina describes the feeling of being overwhelmed by other people through the experience of touch in a situation where a stranger reaches out to shake her hand.

It’s a very panicked feeling. It’s as if you are presented with a box in which you know there’s something inside, and people want you to feel what it is. It’s the same feeling of “ohhh no, something is there!” and you do not know what is in the box or what this sensation is going to feel like […] You do not really know what the person wants, just like you do not know what’s in the box. Like in Diva’s in the Jungle [Danish reality TV program, ed.] where they put a pig’s heart or some chicken in there, and the participants have to stick their hands in there. You do not know what’s in the box. Some people have very soft hands, some feel kind of rough, and some are almost a little wax-like as if their hands are greasy. […] So you do not know what is reaching for you, and you do not know what this person will do with your hand.

Like the descriptions recounted above, Nina communicates the feeling of being overwhelmed through the intense experience of sensory proximity. In the situation, she avoids reciprocating the stranger’s gesture. Instead, her experience concerns the feeling of the hand approaching, closing in, and the preemption of what awaits when it finds her. Her anticipation of the hand’s texture, its possible greasy, wax-like, rough, or soft qualities, engulfs her experience to a point where she cannot respond to the handshake as a meaningful gesture. In her experience, what approaches her is not a handshake but something she compares to raw meat. A shared feature of the descriptions of social situations addressed above is the sense of the sensory surroundings, whether buzzing voices, peripheral movements, or skin texture, closing in, approaching, and becoming experientially invasive.

In the examples recounted above, the experience of the other person as immediately meaningful, which both Husserl and Merleau-Ponty associated with most social encounters, seems to be obstructed by the overwhelming experience of the sensory features of the encounter, such as the texture of skin in Nina’s case or the auditory quality of voices in Johanne’s case. The constant surfacing of these material features of the other’s body as thematic objects of attention renders the other alien and uncanny and amounts to a form of experiential noise or static that interferes with the possibility of social engagement. One might even say that sensory experience introduces a veil of perception that disrupts the immediacy with which the other person appears meaningful.

Furthermore, what permeates the experiences described above is their sensory-affective dimension—social situations and interactions present as chaotic and unpredictable and with a sense of oppressiveness and threat. For example, in the case of Nina’s experience with the stranger, the handshake is experienced as a violent approach of something frightening and unexpected rather than a friendly gesture. Similarly, Johanne’s experience of being engulfed by a sea of noise implied a strong sense of affective alienation from the social situation from which she experientially withdraws when social expectations make it difficult for her to leave physically. In her own words, “I can get so distant and almost isolated from what happens around me because I just shut down.” Thus, sensory experience in autism can disrupt the sense of familiarity with others and the sense of security in social situations.

### Familiarity and Alienation in Autistic Social Experience

According to Køster, the feeling of familiarity with other people is so fundamental to social experience that “like many phenomenologically interesting experiences, it may only become salient through its explicit absence” (Køster, 2021, p: 58). The following descriptions reveal such an absence by presenting experiences of social encounters in which a sense of alienness and opacity permeate the social situation and the other person. Moreover, they reveal how this felt sense of the other disturbs the possibility of social engagement by promoting affective withdrawal and disconnection.

Hanna, a 17-year-old woman with Asperger’s Disorder, describes her response to a social situation, where the soundscape of the family gathering on Christmas Eve feels overwhelming and chaotic:

It is like I’m beginning to shake uncontrollably and cannot sit still. I just want to get out of my body, although I cannot. No matter what I do, I cannot get calm. [Interviewer: And then what do you do?] I try to push it away, but it’s difficult because you hear sounds no matter how much you do not want to hear them. You cannot just shut down your hearing. I get very quiet and shut within myself so I can focus better, and I try to close… or to go into myself and just try to do whatever it takes to be in this situation, and yeah, to create a bubble around myself.

Hanna’s experience points to the important affective dimension of feeling overwhelmed by others by describing the feeling of anxiety and petrification. Her description of a form of emergency shutdown communicates a feeling of disconnection and detachment from the social situation and other people. Below, Helene, a 17-year-old woman with Asperger’s Disorder, expresses a similar sense of disconnection from others in her description of the experience of engaging in eye contact.

It feels very… Not intimate exactly, but something like it. It’s very overwhelming. […] It feels like they can see more. Like, they see me, and I see them, and then I feel insecure about how they see me. Like, how they look back. […] I’m looking at them, and I can see that they are observing me, and that makes me insecure because there is something that I feel like I’m not seeing or something that I do not really know how to, like, see. […] It’s like there is a link missing between my perspective and how others experience it. I’m missing a bridge between the two. There is just a gap. […] There is a bridge between me and the other person, and I think they can cross that bridge, but I cannot do it because there is a gap that does not exist for them.

From this excerpt, it is evident that Helene has difficulty finding the words to describe her experience. She feels as though something is missing in her experience of the other person, something that they can see in her but that she cannot access in them. The sense of familiarity with the other is missing, and what features instead is the feeling of insecurity, anxiety, and disconnection from others, described vividly as the experience of a missing bridge. The feeling of being overwhelmed in eye contact is thus simultaneously characterized by the experience of social detachment and alienation. Expanding on another aspect of coming into contact with strangers, Nina describes eye contact as uncomfortable because of the access seemingly enjoyed by the other person:

It feels weird to sit and look into each other’s eyes. Because I feel that people are kind of looking into… If you are sad, then they will see it. Looking into each other’s eyes seems a little intimate. […] When you are among other people, and you feel sad or stressed, then you avoid eye contact because you can feel that they can see it on your face instantly. […] I get very uncomfortable and conscious when I have that kind of contact. It’s a feeling of shaking, wanting to adjust one’s clothing, and feeling that they see me. I become afraid to look wrong or to make a wrong movement.

In this description, the other person seems to come too close and have too much access, which returns Nina’s awareness to how she looks through the other person’s gaze rather than how they look through hers. Like Helene, Nina experiences a break in the mutuality of social experience manifested through the affective lens of anxiety and discomfort.

The experience of other people as oppressive and unpredictable and of social situations as invasive, chaotic, and claustrophobic run through the various descriptions of social encounters recounted above. Furthermore, this feeling is closely associated with the experience of detachment from and inaccessibility of other people and social meaning. Being sensorially overwhelmed introduces a sense of threat and a desire to move away from rather than toward the world and others. Indeed, a sense of perplexity and petrification seems to have replaced the sense of ease and fluency characterizing social encounters from the perspective of phenomenology. Moreover, these descriptions bear witness to a break in the reciprocity of social experience, which perhaps constitutes the most fundamental feature of phenomenological accounts of social experience (Merleau-Ponty, 1964b; Husserl, 1982).

According to Husserl, the experience of the other person is enabled by a pre-reflective formation of phenomenal unity between the other’s body and one’s own. This process, termed “pairing,” involves what Husserl describes as a “mutual transfer of sense” whereby “this body must forthwith appropriate from mine the sense: animate organism” (Husserl, 1982, p: 113). Pairing thus relies on a sense of familiarity between bodies, a sense of bodies being of the same kind, and is therefore not only a perceptual but also an inherently affective process. The examples presented above speak to this relationship between the presentation of another person in sensory experience and the affective tonality with which that other person appears. Thus, sensory differences in autism destabilize the other person’s appearance as a meaningful expressive unity. With Køster’s phrasing, we can understand autistic sensory experience as destabilizing the sensorium of the other person by transforming the affective tonality of the social encounter and promoting disengagement and withdrawal. In the following, I will explore various processes and practices of restoring this sense of security in and connection to social situations.

### Sensory Urges and Social Deliberations: Strategies of Reconnection

Research participants generally described two ways of dealing with the experience of social interaction. First, in response to the feeling of detachment from one’s surroundings, participants described a strategy of seeking out sensory involvement to provide a sense of presence and focus during stressful experiences of a chaotic and threatening sensory environment. This strategy implied an inherent ambiguity, as sensory experience both instituted and resolved the feeling of being overwhelmed in social situations. Second, experiences of others as opaque, alien, and of social detachment were handled by relying on thinking as an approach to social understanding. This strategy provided a feeling of having an access point, a sense of footing, and a point of orientation in an inherently disorienting encounter with another person.

In her description of whirling dancers, Nina communicates how she also experiences the feeling of being overwhelmed by others as an urge to look at them:

You can see it out of the corner of your eye, and you want to look because it takes up your attention, but you also know that you are sitting and talking with someone. Then there is perhaps someone at another table getting up, and then you want to look at that also.

Being overwhelmed is simultaneously a feeling of being drawn in or disturbed by the surroundings. To relieve this disturbance, Nina describes using headphones with music playing in one ear to shut out what is experienced as intrusive and as an anchor securing her sense of presence in the situation.

I tend to follow conversations around me, and I cannot shut them out. [If I listen to music, ed.], then my brain does not concentrate on those conversations but on the music. If I start to feel anxious and there are many people, then I can just concentrate on the music […] as a way to get away if anything happens. […] I tend to put some music in my ears because it works as an automatic wall being put up. The bass will thump a little in your ears or vibrate a little. […] Otherwise, I get very restless about not being allowed to look.

The sound and vibration of the music thus function as ways of calming Nina’s restlessness and, by experientially separating her from the chaotic surroundings, provide ways to fasten and center her attention on the interaction in which she is engaged. Line, a 17-year-old woman with Asperger’s Disorder, describes how a disciplined focus on single aspects of the visual environment can ease this impulse to follow movements or sounds around her. In the following, she describes a dinner party with her parents and their family friends:

Everything is just turning and turning around you… And I actually have a sort of urge to really know where everything comes from. Every time I sense movement, I look to see what it is, or if I hear a sound, I look to see where it comes from. […] I try to focus on something different. It can be looking out the window or looking down at a… the glass of water in front of me. […] I do not know… Maybe it makes sense somehow, so I can calm down a little by focusing on some object and say, “okay, now it is this thing, which is important.”

In this way, Line willfully absorbs herself in a particular aspect of the sensory world to navigate the chaotic sensory environment represented by the dinner party. The glass of water standing in front of her at the dinner table acts as a sensory anchor maintaining her presence in these overwhelming visual and auditory surroundings. She forces the object to appear meaningful and stand out in the muddle and chaos around her by deliberately focusing on the glass. Helene describes a similar strategy in the context of touch:

I often seek stimuli when I come home to my parents. They know that they should hug me hard. […] I’ve started to seek out being squeezed or pressed by my parents when I’ve had a long day. […] I get very overwhelmed in my body and in my head, and then the more hard pressure somehow grounds me. Because everything feels like static electricity, I do not know how to explain it, you know, like if the TV flickers.

Seeking out touch as hard, constant pressure relieves Helene of the static and flickering sense of touch she describes as part of her sensory issues. Like Line’s visual absorption in the glass of water, Helene immerses herself in the experience of a clear, demarcated, and steady touch rather than the buzzing or fluttering tactile impressions that she elsewhere refers to as feeling “like bees.”

Nina, Line, and Helene describe this deliberate grounding and centering of dispersed attention by seeking out sensory experiences in different ways. While Nina describes an auditory strategy of listening to music, Line describes a visual strategy of focusing on an object in her immediate surroundings, and Helene describes a tactile strategy of seeking firm pressure. In their descriptions, such practice of sensory seeking allows an experiential anchor turning them away from a chaotic and unpredictable situation and toward a sense of presence to the social interaction in focus.

Research participants described another strategy of using reflective attention and deliberate reasoning as an approach to social interaction and understanding the social expressions of others. For example, Nina describes the following situation, where she is asked directions in public transport:

When someone approaches me like that, I just sit there with cold sweats. I do not know what to answer and fumble with the words. I feel somewhat strange or off and very conscious about my movements. I just feel strange. […] It is as if the world stops. I feel a chill and get all stiff in my body. From feeling quite light and free, I suddenly feel trapped in a cage and have to answer […] “Normal people,” quote-unquote, would probably just answer her because they are totally used to automatically reading facial expressions, tone of voice, etc. But if I have to answer her, then I first have to figure out what her face is telling me, what her body posture is like, what is her tone of voice, what she is actually saying, what is the mood in the situation. Like, is she looking angry, is she angry, does she seem angry, or is she surprised, happy, etc.? All of these things have to be turned around in my head, and people expect an answer fairly fast, so If you do not answer within, say, 30 s, people will start to question whether you even heard them.

Faced with the stranger’s question, Nina describes a feeling of perplexity, insecurity, and motionlessness, almost as if she were a deer caught in the headlights. She describes the prospect of being put on the spot while incapable of answering within the expected timeframe as particularly stressful. Rather than what she describes as an “automatic reading,” she goes through a laborious process of thinking through individual aspects of the other’s bodily expressions one at a time. In this systematic way, Nina works out what is expected of her in this social situation by drawing on analytic and reflective resources. Like the practice of sensory seeking described above, this strategy applies an active approach to something that would typically be passive or intuitive. Both represent practices of deliberately and actively seeking out social meaning in an overwhelming social situation and thus reconnecting to other people when faced with the experience of their unfamiliarity and unpredictability. In the case of thinking through social interaction processes, it represents a practice of deliberately and laboriously seeking out social meaning through an analytic approach. In the case of sensory seeking, it functions as the deliberate attempt to anchor oneself in single aspects of the sensory surroundings to provide a sense of presence despite the experience of a chaotic and fragmented world.

To understand these strategies better, we can return to Merleau-Ponty’s analysis of the relation between object and horizon in perceptual experience. His idea that “it is necessary to suspend the surroundings in order to see the object better” and that “to see the object is to plunge into it” (Merleau-Ponty, 2012, p: 70) helps understand sensory seeking as a way to deliberately plunge into objects. Sensory absorption, whether visual, tactile, or auditory, represents ways of forcing objects to stand out: to tear away figure from ground and thus re-establishre-establish a relationship between foreground and background in experience. In this way, perceptual significance is sought by actively and intentionally singling out particular objects or aspects of the sensory environment. A perceptual grip is thus re-established in a situation that would otherwise appear as a jumbled confusion of fragmented sensory impressions.

As the above analysis has revealed, an essential aspect of coping with the sensory density of social situations is using reflective attention toward something that is typically an intuitive and automatic process. In everyday neurotypical experience, the world appears as a meaningful network of objects and relations whose situationally and contextually relevant aspects effortlessly reveal themselves. In this case, social meaning is wrenched out through an effortful and laborious process robbed of the sense of ease and fluency that phenomenological accounts often ascribe to social understanding and interaction. In the following, I will discuss the implications of this relation between sensory and social experience in autism for discussions on the role of practice, grip, and compensation in disability and psychopathology.

## Discussion

The diverse experiences recounted above revealed how social encounters for autistic persons can manifest as sensorially disturbing and chaotic events and how other people appear unfamiliar and threatening in this context. Moreover, in response to the sense of detachment and disengagement from the social world experienced by autistic persons, strategies of reconnection were mobilized that actively sought out perceptual meaning in an inherently strange and unpredictable situation. This analysis highlights both the role of receptivity and responsivity in how other people appear in social experience (Køster, 2021) and the role of practice and activity in the experience of perceptual and social meaning (Salamon, 2012).

Køster emphasizes how the felt sense of the other is a phenomenological structure characterized by reciprocity between what can be termed a style of the perceiver and a style of the perceived.

Although the expressive style of the other has a definite autonomy and specificity, it cannot be regarded in isolation from the ontogeny and habituated style of perception of the perceiver. In the sensorium of the other these two aspects intertwine intercorporally (Køster, 2021, p: 64).

In this paper, I have explored how habituated perceptual styles in autism destabilize and fragment the sensorium of the other and transform the affective tonality, or felt sense, with which the other appears. The idea that a deep sense of familiarity with others is fundamental to our experience of the social world echoes through this analysis, in this case, by demonstrating how such a sense of familiarity can be disrupted. Concerning the reliance on direct and pre-reflective access to social meaning in phenomenological accounts, this analysis emphasizes how such access is negotiated with the particular form of embodiment enjoyed by the perceiver. In this case, the features of autistic sensory experience introduced an aspect of mediation in social experience, thus pointing to the intuitive givenness of social meaning as a privilege of the able-bodied.

In addition to the importance of perceptual style in social encounters, this analysis emphasizes the importance of practice and activity in the experience of social meaning. In her analysis of maximal grip, Gayle Salamon argues that disabled embodiment reveals a fundamental structure in the relation between self and world, namely that our enmeshment with the world is not characterized by dexterous mastery and expertise (Salamon, 2012, p: 244). Where Hubert Dreyfus characterizes maximal grip as the body’s fluent ability to bring the world closer to an optimal gestalt, Salamon emphasizes how embodiment should be understood as something that can be thrown out of balance and vulnerable in the encounter with the world and other people (Salamon, 2012, p: 250). Through reading two disability memoirs, Salamon identifies an aspect of grip what could be termed practice.

Grip is deployed in both cases not as a way of enmeshing seamlessly with the world, but as a means of methodically composing the body as a substitute for an unthought, and now foreclosed, enmeshment with the world. Grip is the use of deliberate action to order the self as a compensation for lost bodily intentionality (Salamon, 2012, p: 248).

In Salamon’s analysis of arthritic embodiment, grip refers to a methodical “taking a grip” on one’s body by testing and checking its flexibility, painfulness, and capacity before engaging in a task. This idea of deliberation in the relationship between self and world is helpful in understanding autistic strategies of reconnection as practices of reestablishing a perceptual grip on the social situation. The practice of actively pursuing sensory stimulation in social situations is a way to gain a sense of footing in and grip on the social situation by forcing the world to stand still in order to see it better. These practices thus act as bodily auxiliaries that facilitate a sense of engagement with an otherwise chaotic and unpredictable sensory and social environment, thus emphasizing the possible role of activity in maintaining a sense of intuitive grasp of social meaning.

The role of practice and activity in autistic social experience invites further consideration of how such practices may be supported and facilitated to strengthen autistic persons’ experiences of social connectedness. For example, the strategy of seeking out sensory stimulation to maintain a sense of presence in social situations calls for an approach that takes seriously the role of objects and the material environment in facilitating social encounters. As is described both in the historical literature and the current diagnostic guidelines, autism is characterized by abnormalities in the use of objects and the relation to the physical environment (Kanner, 1943; World Health Organization, 2018). Recent debates in psychology and cognitive science concerning the role of objects in social and psychological development have motivated an interest in how the use of objects may facilitate processes of social interaction and communication in autism (Iannaccone et al., 2018; Williams et al., 2018; Manzi et al., 2020). In the field of psychology, a socio-material and ecological perspective highlights the constitutive function of objects and artifacts in psychological and social development, and in the field of cognitive science, enactive, embedded, and material theories of cognition have emphasized the role of interactive context and materiality in social interaction and cognition (Gibson, 1979; de Jaegher and di Paolo, 2007; Malafouris, 2013; Pedersen and Bang, 2016; Rietveld et al., 2018). Thus, an important avenue to pursue further is, on an empirical level, how social aspects of autism relate to aspects typically considered a social, such as a typical use of objects, insistence on sameness, or repetitive behaviors, and, on a theoretical level, how intersubjectivity relates to the material environment [see Boldsen (forthcoming) for further exploration of these issues].

In this article, I have presented descriptions of autistic social experience that emphasize how the surrounding (social and physical) world appears as a strange, overwhelming, and chaotic place. Taking this relational view of social experience seriously implies considering autistic social behaviors as attempts to navigate and meaningfully respond to this uninhabitable world of experience. It is well known that autistic persons employ a range of strategies to function in neurotypical social contexts, such as applying learned rules to social interactions or other ways of masking autistic difficulties (Livingston et al., 2020). Such compensatory strategies are often described as of a cognitive nature and directed toward compensating for theory of mind difficulties in social situations (Livingston et al., 2019). A related point is expressed in the phenomenological literature on autism, where Dan Zahavi and Josef Parnas argue that autistic individuals resort to an intellectually driven approach to social encounters as a way to compensate for “a lack of an immediate, pre-reflective, or implicit understanding of the meaning of social interaction” (Zahavi and Parnas, 2003, p: 67). Here, Zahavi and Parnas build on insights from schizophrenia research, where hyperreflexivity is defined as an overreliance on reflective attention toward oneself and one’s surroundings to compensate for a diminishment of the pre-reflective grasp of meaning (Sass and Parnas, 2003; Fuchs, 2010). However, rather than representing such strategies as pathological attempts to compensate for a lack, I think they bear witness to an important sensory and perceptual aspect of compensation, which casts autistic social difficulties and reparatory practices as profoundly world-involving. Furthermore, phenomena such as masking emphasize the disabling and discriminatory effects of social norms and expectations in different societal contexts (Radulski, 2022). An interesting avenue to pursue considering the current study is developing research approaches sensitive to both experiential and societal features of autism that would help address the question of how sensory experience relates and responds to social structures, norms, and expectations in concrete social contexts and interactions.

Autism painfully demonstrates how perception is a continuous project of negotiating the reversibility of gripping and being in the grip of the world, of moving and being moved by the world (Salamon, 2012; Thoma and Fuchs, 2017). This emphasizes how autism can be understood as an exceptional style of being rather than an expression of underlying impairment. Joel Krueger, characterizes autism as “a felt sense of being bodily and affectively out-of-sync with neurotypical spaces not set up to accommodate non-neurotypical styles of being in the world” (Krueger, 2021, p: 3). This mobilization of the notion of style to describe psychopathology draws attention to the qualitative differences between various modes of inhabiting the world rather than a normative understanding of one mode being an impoverished version of another. This understanding of autism ultimately invites phenomenology to interrogate not only typical but also diverging modes of being (see Carel, 2021) and consider how they may productively expand and nuance basic ideas and analyses of how we understand and relate to one another bodily and intersubjectively.

## Conclusion

In response to an increasing interest in the bodily and experiential aspects of autism from the perspectives of both autism research and phenomenology, this paper has explored the relationship between sensory and social experience in autism. By drawing on qualitative data from an ongoing phenomenological study on social experience in autism, I have argued that sensory differences in autism affect the way social encounters appear in autistic experience on a pre-reflective level. Through an analysis informed by phenomenological accounts of intersubjectivity and perceptual experience, three aspects of how sensory differences affect autistic social experience were identified.

First, the diverse social experiences recounted by research participants revealed how social encounters for autistic persons can manifest as sensorially disturbing and chaotic events. These events appeared with a blurred distinction between the thematized and the tacit in perception, creating an experience of the social situation as experientially invasive and unpredictable.

Second, in this sensorially imminent and approaching world, the embodied expressions of the other appeared alien and with an affective tonality of threat, disrupting the sense of familiarity with others and security in social situations. Thus, sensory differences in autism destabilize the appearance of the other person as a meaningful expressive unity, leaving an experience of opacity and inaccessibility of social meaning that disturbs the possibility of social engagement.

Third, participants described practices of deliberately and actively seeking out social meaning in an overwhelming social situation and, in this way, reconnecting to other people when faced with the experience of their unfamiliarity and unpredictability. One strategy relied on sensory absorption, whereby an experiential distinction between figure and ground was established by actively pursuing perceptual significance. Another strategy applied reflective resources to understand the embodied expressions of others to a sense of footing in a deeply disorienting encounter with another person.

This analysis contributes to emerging phenomenological approaches to autism by pointing to specific ways in which sensory differences are implicated in autistic disturbances in bodily being-with-others and thus stresses the importance of looking at embodied intersubjectivity in terms of its sensory dimensions. By approaching social encounters as sensory and perceptual events, social experience is emphasized as an inherently and irreversibly world-involving phenomenon. This highlights the importance of looking at how contextual and situational elements affect the manifestations of autism and further on the extent to which sensory differences affect autistic experience of the world and others. In addition, the account of sensory and social experience forwarded in this paper pointed to various practices of regaining a sense of perceptual and social grip as forms of bodily auxiliary that mediated a sense of presence and footing in an otherwise chaotic and unpredictable social world. This emphasized the potential role of activity in maintaining a sense of intuitive grasp on social meaning and indicates an important avenue for future research in exploring how such activity may help understand other aspects of autistic social experience and practice.

## Data Availability Statement

Due to confidentiality agreements and ethical concerns, supporting data cannot be made openly available. Questions regarding the datasets should be directed to SB (boldsen@ruc.dk).

## Ethics Statement

The study was reviewed by the Danish Committee System on Health Research Ethics. Written informed consent was obtained from the participants and minor participants’ legal guardians for the publication of data included in this article.

## Author Contributions

The author confirms being the sole contributor of this work and has approved it for publication.

## Conflict of Interest

The author declares that the research was conducted in the absence of any commercial or financial relationships that could be construed as a potential conflict of interest.

## Publisher’s Note

All claims expressed in this article are solely those of the authors and do not necessarily represent those of their affiliated organizations, or those of the publisher, the editors and the reviewers. Any product that may be evaluated in this article, or claim that may be made by its manufacturer, is not guaranteed or endorsed by the publisher.

## Footnotes

^1^Part of this starting point is also embracing identity-first language to acknowledge how autism is increasingly being understood by those diagnosed with autism as an integral part of one’s identity and being rather than reproducing problematic assumptions of autism as a curable disease or error to be corrected (Vivanti, 2020; Botha et al., 2021). Although person-first language usually intends to emphasize the value and humanity of the person, in the autism community and disability movement, it is often taken to imply that “autism is entirely separate from what gives him or her value and worth,” and ultimately that “it would have been better if he or she had been born typical” (Brown, 2011).

^2^One aspect of this push for recognition is the autism community’s movement toward the reappropriation of autistic bodily expressions, such as repetitive movements and self-stimulatory behaviors (“stimming”), which historically have been depicted from a behaviorist perspective as something to be eliminated (Boyd et al., 2012).

^3^The advent of these embodied and phenomenological approaches to autism relates to a parallel movement in cognitive science, where second-person approaches are gathering momentum. By promoting the idea that social understanding emerges from and is conditioned by concrete and embodied interaction (de Jaegher and di Paolo, 2007; Schilbach et al., 2013; Ferrer de Luna, 2019), these approaches contribute to developing an embodied and relational approach to autism (Gallagher, 2004; de Jaegher, 2013, 2021).

^4^The view that mental states are directly available in perceptual experience bears on the idea that intentions and emotions are concretely embodied in expressive behavior. Seeing, hearing, or feeling another person’s bodily expressions thus present their emotions or intentions directly, that is, without mediation from reflection, inference, or metarepresentation. This phenomenological understanding of perception and social experience has recently gained increased attention in 4E approaches to cognitive science under the heading “direct social perception” (Spaulding, 2015; Krueger, 2018). One potential challenge for such an approach to social cognition is accounting for how social interaction and understanding develop throughout life and become smoother with time. Here, it is fruitful to take a closer look at the idea of perception implied in this view. Perception is not a passive, observatory stance through which we receive and subsequently process sensory input. Rather, perception is inextricably tied to bodily activity. In the case of social perception, experiencing and understanding the other’s expressive behavior is an interactive process. Thus, the complexity and nuance of social understanding develop and mature as social interaction also becomes more complex. For additional discussion, see Vincini and Gallagher (2021).
